# P064: Direct identification of vancomycin-resistant enterococci from selective enrichment broth by mass spectrometry

**DOI:** 10.1186/2047-2994-2-S1-P64

**Published:** 2013-06-20

**Authors:** B-M Woo, H Lee, S-I Joo, E-C Kim

**Affiliations:** 1 Department of Laboratory Medicine, Seoul National University Hospital, Seoul, Korea, Republic Of

## Introduction

It is mandatory in South Korea that patients and carriers, from whom vancomycin-resistant enterococci (VRE) are detected, should be isolated from the other patients. The rapid detection of VRE is essential to prevent the dissemination of VRE. We determined the possibilities of direct identification of VRE from selective enrichment broth by mass spectrometry for the shortening of turn-around-time.

## Methods

During the one-month period of VRE outbreak investigation, 50 rectal swabs were incubated into enterococcal broth containing 6 μg/mL of vancomycin at 37°C for 24 hours. For the rapid identification of VRE, total 50 pellets obtained after the centrifugation of one mL were applied on mass spectrometry. The results of the mass spectrometry were compared to those of standard culture using chromogenic agar for the detection of VRE.

## Results

Among total 39 VRE isolated by standard culture, 32 were *E. faecium* only, and seven were mixed with both *E. faecium* and *E. faecalis*. By mass spectrometry total 33 VRE were identified of which 26 were *E. faecium* only and six were mixed with both *E. faecium* and *E. faecalis*, and one was *E. faecalis* only. Total 17 VRE-negative cases by mass spectrometry were four no-peak-found, four no-reliable, two *Lactobacillus*, four *E. gallinarum*, two *E. avium*, and one *Pediococcus*. Compared to chromogenic agar method, the sensitivity and specificity of mass spectrometry method were 84.6% and 100.0%, respectively.

## Conclusion

Direct identification of VRE from selective enrichment broth by mass spectrometry may be helpful to shorten the turn-around-time. The mass spectrometry method can detect VRE one day earlier than the conventional method.

## Disclosure of interest

None declared

